# Chronic Antipsychotic Treatment Modulates Aromatase (CYP19A1) Expression in the Male Rat Brain

**DOI:** 10.1007/s12031-019-01307-x

**Published:** 2019-04-09

**Authors:** Katarzyna Bogus, Artur Pałasz, Aleksandra Suszka-Świtek, John J. Worthington, Marek Krzystanek, Ryszard Wiaderkiewicz

**Affiliations:** 1 Department of Histology, School of Medicine in Katowice, Medical University of Silesia, ul. Medyków Street 18, 40-752 Katowice, Poland; 2 Division of Biomedical and Life Sciences, Faculty of Health and Medicine, Lancaster University, Lancaster, LA1 4YQ UK; 3 Department and Clinic Psychiatric Rehabilitation, School of Medicine in Katowice, Medical University of Silesia, ul. Ziolowa 45/47, 40-635 Katowice, Poland

**Keywords:** Aromatase, Brain, Olanzapine, Clozapine, Neuroleptics

## Abstract

Antipsychotic drugs, known as the antagonists of dopaminergic receptors, may also affect a large spectrum of other molecular signaling pathways in the brain. Despite the numerous ongoing studies on neurosteroid action and regulation, there are no reports regarding the influence of extended treatment with typical and atypical neuroleptics on brain aromatase (CYP19A1) expression. In the present study, we assessed for the first time aromatase mRNA and protein levels in the brain of rats chronically (28 days) treated with olanzapine, clozapine, and haloperidol using quantitative real-time PCR, end-point RT-PCR, and Western blotting. Both clozapine and haloperidol, but not olanzapine treatment, led to an increase of aromatase mRNA expression in the rat brain. On the other hand, aromatase protein level remained unchanged after drug administration. These results cast a new light on the pharmacology of examined antipsychotics and contribute to a better understanding of the mechanisms responsible for their action. The present report also underlines the complex nature of potential interactions between neuroleptic pharmacological effects and physiology of brain neurosteroid pathways.

## Introduction

Aromatase (estrogen synthase, CYP19A1), a tissue-specific product of cytochrome P450*cyp19a* gene located in chromosome 15, is a key mammalian microsomal enzyme in the process of the conversion of androgens to estrogens (Brocca and Garcia-Segura 2018; Roselli et al. 2009; Stoffel-Wagner 2001). The activation of both appetitive and consummatory sides of male sexual behavior are triggered by testosterone aromatization. A distinct aromatase expression is found in the gonads and several other organs including the brain. In rats, the presence of aromatase has been detected exclusively in the brain, testes, and ovaries (Kato et al. 1997). Expression of aromatase is generally limited to adult neurons (Garcia-Segura et al. 2003), it does not normally occur in the glial cells except in reactive astrocytes (Pedersen and Saldanha 2017, Azcoitia et al. 2003; Garcia-Segura et al. 1999) and radial glia (Xing et al. 2016) following neurotoxic and mechanical injury. Aromatase activity is modulated by steroid-related transcriptional changes but also more rapidly by phosphorylation cascades. Sexual activity of both male and female rodents may also affect brain aromatase activity, probably through glutamatergic signaling (Balthazart 2017; de Bournonville et al. 2017; Antaramian et al. 2015). Recent evidence suggests a role for brain aromatase in the development of serotoninergic pathways in lower vertebrates (Ulhaq and Kishida 2018). Since the function of steroid sex hormones is relatively well studied at the level of the reproductive glands, their physiological roles in brain structures are still insufficiently explained. In the various regions of the human brain, such as the frontal and temporal lobes, hippocampal formation, and hypothalamus, a distinct, sex-independent aromatase mRNA expression has been detected (Stoffel-Wagner 2001). Interestingly, some of these structures show a diverse pattern of estrogen receptor isoform expression, e.g., in the hypothalamic nuclei ERα predominates, whereas in the hippocampal and cortical areas, ERβ is most abundant (Simpson et al. 2002). Hippocampal aromatase seems to be necessary to induce long-term potentiation (LTP) and maintain neural plasticity (Azcoitia et al. 2017, Vierk et al. 2012). On the other hand, a recent study revealed a sex-dependent involvement of aromatase in the regulation of synaptic functions in the rat basolateral amygdala, a main center of fear responses, where the neurosteroid 17β-estradiol (E2) is synthesized. It may be important for the understanding of sex differences in the course of some neuropsychiatric diseases, e.g., bipolar disorder or anxiety (Bender et al. 2017). Moreover, there are suggestions that the neuropsychiatric manifestations of brain aromatase blockade, e.g., during post-menopausal pharmacotherapy and subsequent inhibition of central estrogen synthesis, could be further associated with modulations of monoaminergic pathways in the prefrontal cortex and hippocampus, the core structures involved in mood learning and memory processes (Kokras et al. 2017; Aydin et al. 2008).

Brain-derived steroids, also known as neurosteroids, may be synthesized by both neurons and glial cells (Benarroch 2007). Numerous reports show that they act as neurotransmitter-like factors as they are released at precise places within neural populations and can regulate very quickly both cognitive and behavioral functions (Balthazart and Ball 2006; Dewing et al. 2007; Saldanha et al. 2011; Remage-Healey 2014). Neurosteroids regulate NMDA and GABA_A_ receptor functions acting as potent allosteric modulators of the ionic channel molecules (Saldanha et al. 2009; Benarroch 2007). They also affect opioid sigma receptors. Interestingly, changes in neurosteroid signaling may contribute to the pathogenesis of anxiety and depression; furthermore, selective serotonin reuptake inhibitors (SSRI) may act at least in part via the restoration of neurosteroid function (Longone et al. 2011). A potential role of brain neurosteroids in the pathogenesis of schizophrenia is also postulated (Shulman and Tibbo 2005). Several medications known as the aromatase inhibitors, such as aromasin, anastrozole, or letrozole, can block estrogen synthesis through the direct suppression of their conversion to androgens (Czajka-Oraniec and Simpson 2010). Noteworthy, both estrogens and their precursors seem to have neuroprotective properties (Saldanha et al. 2009; Stoffel-Wagner 2001).

Olanzapine, an atypical neuroleptic, has an affinity to various brain receptors including dopaminergic D_1_-D_5_, serotoninergic 5-HT_2A/2C_, 5-HT_3_, 5-HT_6_, α_1_-adrenergic, muscarinic M_1_-M_5_, and histaminergic H_1_.This antipsychotic drug reduces positive schizophrenia symptoms through a decrease of dopaminergic neuron activity, mainly in the mesolimbic tract, but it exerts only a minor effect on the striatal motor systems (Harvey et al. 2016; Leucht et al. 2013). The metabolism of olanzapine occurs in the liver where two cytochrome P450 isoforms CYP1A2 and CYP2D6 proceed drug biotransformation into N-desmethylolanzapine and 2-hydroxyolanzapine respectively (Prior and Baker 2003). The final, inactive, olanzapine metabolite 10-N-glucuronide does not cross the blood-brain barrier (Aravagiri et al. 1999).

Clozapine, another dibenzodiazepine-derived atypical neuroleptic with high inhibitory affinity toward dopaminergic D4 receptors, is a less potent dopamine D2 blocker than typical antipsychotics. Clozapine may also inhibit glutamate reuptake, through a decreased expression of both neuronal and astrocytic glycine transporters: EAAT3 and EAAT2 respectively (Krzystanek et al. 2015). It may also inhibit the neuronal D-aspartate oxidase (DDO) activity that increases glutamate release in the mouse brain (Sacchi et al. 2017). The two main products of clozapine biotransformation via liver microsomal isoforms CYP1A2 and CYP3A4 are desmethylclozapine (norclozapine) and N-oxide clozapine (Spina et al. 2003; Linnet and Olesen 2000; Eiermann et al. 1997).

Haloperidol, a typical but still commonly administered D_2_ receptor antagonist, effectively reduces psychosis suggesting impaired dopaminergic signaling is a key mechanism of positive schizophrenia symptoms (Gass et al. 2013). However, it shows a wide spectrum of unfavorable side effects including akathisia, tardive dyskinesia, neuroleptic malignant syndrome, and heart rhythm disturbances (Leucht et al. 2013; Stracina et al. 2015). Noteworthy, haloperidol is a non-specific neuroleptic with affinity to numerous receptors, including dopamine D2, serotonin 5-HT2, α-adrenergic, and σ-opioid receptors (Cobos et al. 2007; Roth et al. 1998). Haloperidol undergoes biotransformation, mainly oxidative dealkylation by CYP3A4 and CYP2D6 isoforms. An increase of the serum concentration of co-administered drugs, known as the substrates for CYP2D6, was reported suggesting a role of haloperidol metabolites in enzyme inhibition (Shin et al. 2001). Interestingly, brain CYP2Ds may alter some side effects of haloperidol and other neuroleptics metabolized by these isoforms (Miksys et al. 2017).

A number of findings revealed that aromatase expression as well as neurosteroid synthesis may be significantly modulated by various neuropsychiatric medications including antidepressants and benzodiazepines (Chen et al. 2016; Schüle et al. 2011; Longone et al. 2011; Niwa et al. 2008, Pinna et al. 2006). However, very little is known about effects of neuroleptic administration on the aforementioned processes. The aim of the study was to comparatively determine, for the first time, whether long-term treatment with both atypical (olanzapine, clozapine) and typical (haloperidol) antipsychotic drugs affects aromatase expression in the rat brain.

## Materials and Methods

Adult (5 months old, 210–240 g) male Sprague-Dawley rats from the Medical University of Silesia Experimental Centre were housed at 22 °C with a regular 12/12 light-darkness cycle with access to standard Murigran chow and water ad libitum. All experimental procedures were approved by the Local Bioethical Committee at the Medical University of Silesia (agreement no. 36/2012) and were conducted in a manner consistent with NIH Guidelines for Care and Use of Laboratory Animals. Four groups of animals (*n* = 5) had received respectively control vehicle, olanzapine (10 mg/kg/day), clozapine (20 mg/kg/day), and haloperidol (1 mg/kg/day), by intraperitoneal injection for 28 days. Three hours after the last drug administration, rats were quickly anesthetized with isoflurane and sacrificed. Their brains were removed and then prepared for further molecular procedures.

Brain hemispheres were homogenized immediately after isolation using an ultrasound homogenizer (Heildolph DIAX 900, Germany). Total mRNA was extracted via phenol-chloroform method using Trizol™ as previously described. Collected mRNA samples were transcribed into cDNA during incubation in a buffered solution of reverse transcriptase MMLV-RT with RNAsin, oligo-dT, and a mix of nucleotides at 42 °C for 60 min using DNA Thermal Cycler 480 (Perkin Elmer Inc., Waltham, MA) After that, quantitative real-time PCR reaction (qPCR) was performed by FastStart SYBR Green Master mix (Roche) in a Light Cycler 1 96 (Roche). Thermal cycler 32 rounds: 1 min at 94 °C, 1 min at 62 °C, and finally 90 s at 72 °C. Beta-2-microglobulin (B2m) was chosen as a standard internal housekeeping reference gene. cDNA was amplified using the following primers (Sigma, Life Science): aromatase CYP19A1 (126 bp): Forward: 5′–TAAAAGATGGCACACAAAGAGTGC, Reverse: 5′–ACCGAGGTTACCTGGATCTGC; B2m: F: 5′-CGAGACCGATGTATATGCTTGC, R: GTCCAGATGATTCAGAGCTCCA. All qPCR data were analyzed using the comparative Cq method. Finally, the formula 2^−ΔΔCq^ was used to achieve relative quantitation of mRNA expression. Additionally, the end-point RT-PCR method was also used to confirm aromatase mRNA expression. The PCR reaction was performed in a Peltier Thermal Cycler PT-200 (MJ Research Inc., Watertown, MA) with the same CYP19A1 primer for 32 rounds: 1 min at 94 °C, 1 min at 65 °C ,and finally 90s at 72 °C. Glyceraldehyde phosphate dehydrogenase was an internal housekeeping reference gene: GAPDH: Forward: 5′–GTGAACGGATTTGGCCGTATCG′, Reverse: 5′–ATCACGCCACAG CTTTCCAGAGG-3. Products of PCR amplification were separated on a 2% agarose gel, visualized with ethidium bromide, and photographed in a UV light chamber. Semi-quantitative densitometric analysis was performed with the use of OneDScan software (Scanalytics). The results were expressed as relative (vs. GAPDH) integrated optical density (IOD) showing relative gene expression.

The second half of brain hemispheres were homogenized (Potter-Elvehjem system) in 0.25 M sucrose in Tris/HCl (pH 7.4). The homogenate was centrifuged at 10,000×*g* for 20 min and then supernatant was centrifuged again at 105,000×*g* for 1 h to sediment the microsomal fraction that was suspended in 20% glycerol in phosphate buffer. Microsomal proteins were separated using SDS/polyacrylamide gel electrophoresis (Biorad) and transferred onto nitrocellulose membrane (Millipore). Aromatase was detected with rabbit anti-rat aromatase polyclonal primary antibody (Abcam, AB18995). The secondary antibody was goat anti-rabbit IgG conjugated with alkaline phosphatase. BCIP/Nitrotetrazolium Blue was used to visualize the enzyme activity. Immunoblots were quantified using One-D-Scan Gel Analysis program (Scanalytics). The results were expressed as integrated optical density (IOD) showing the relative protein expression.

Statistical analysis was performed using Statistica 10 (Systat software). Gaussian distribution and variance homogeneity were estimated with Shapiro–Wilk and Levene’s tests. The results were then statistically analyzed using one-way ANOVA and HSD Tukey’s tests. Differences were considered statistically significant at *p* < 0.05.

## Results

**Fig. 1:**
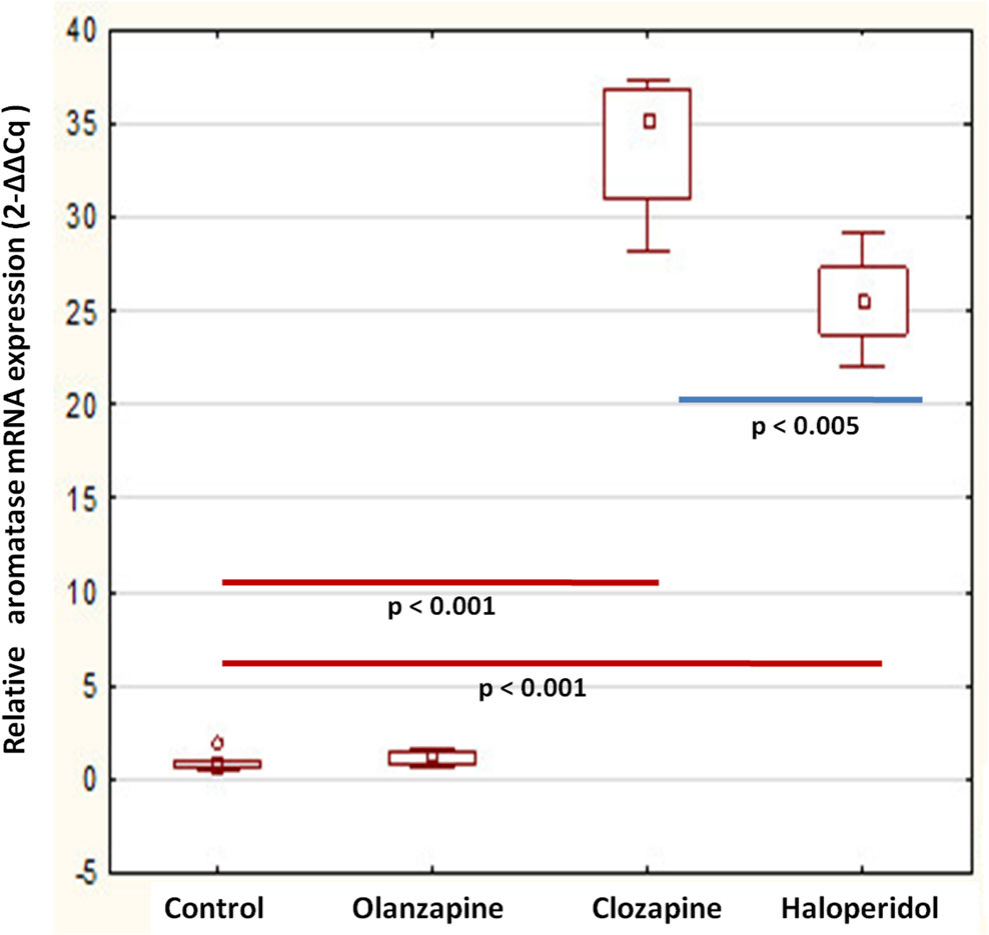
Quantitative real-time PCR results of relative aromatase mRNA expression levels in the rat brain. Obtained results were normalized to GAPDH housekeeping reference gene. Data are presented as mean 2^−ΔΔCq^. One-way ANOVA followed by Tukey’s HSD post-hoc test was used for statistical analysis (experimental group vs control). *p* ≤ 0.05 is considered as statistically significant

**Fig. 2:**
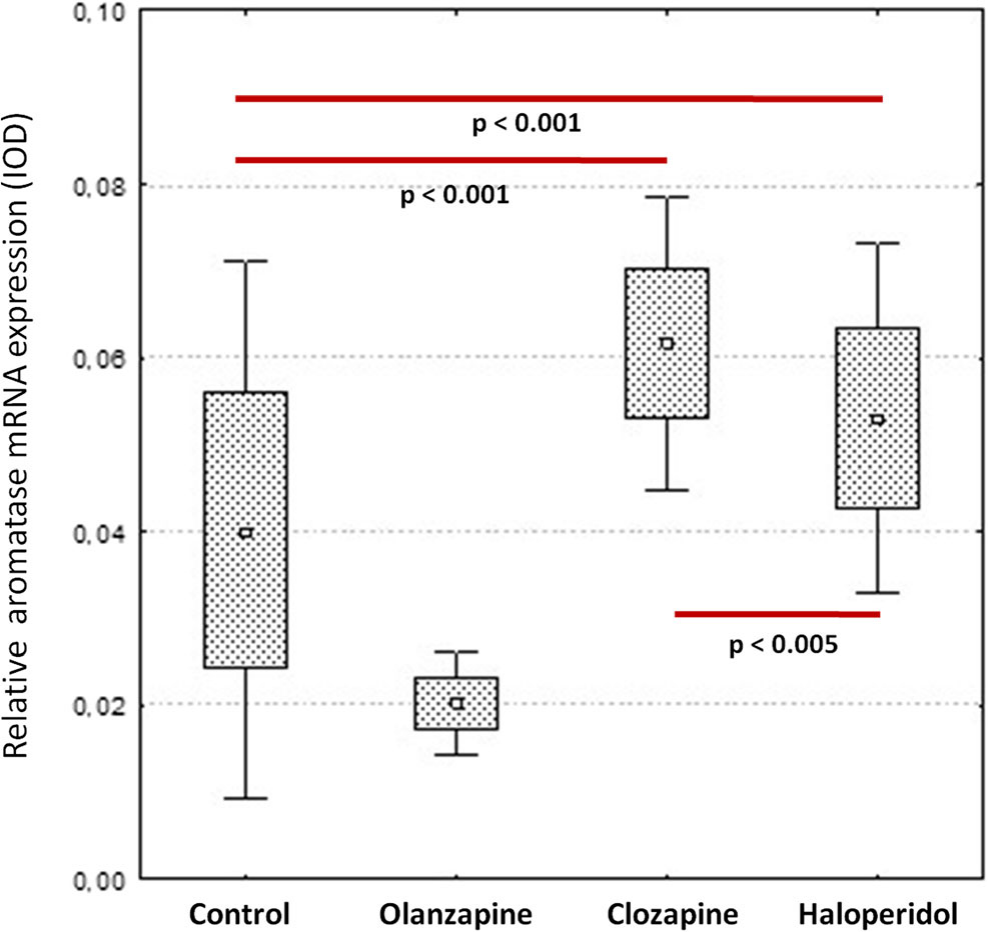
Semi-quantitative RT-PCR results of relative aromatase mRNA expression levels in the rat brain. Obtained results were normalized to GAPDH housekeeping reference gene. Data are presented as mean integrated optical density (IOD). One-way ANOVA followed by Tukey’s HSD post-hoc test was used for statistical analysis (experimental group vs control). *p* ≤ 0.05 is considered as statistically significant

**Fig. 3:**
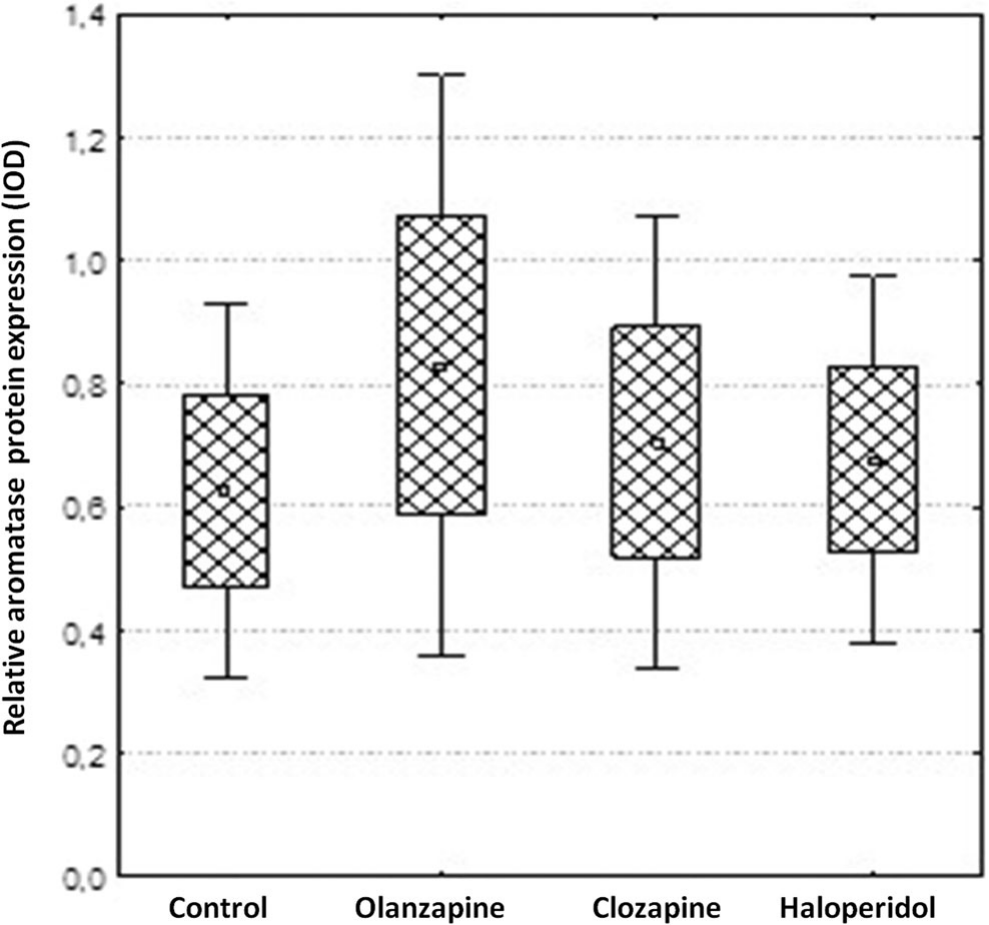
Relative aromatase protein levels in the rat brain. Data are presented as mean integrated optical density (IOD). One-way ANOVA followed by Tukey’s HSD post-hoc test was used for statistical analysis (experimental group vs control). *p* ≤ 0.05 is considered as statistically significant

The present real-time PCR study shows that whole brain aromatase mRNA expression was significantly increased after long-term treatment with clozapine (33.94 ± 4.15) and haloperidol administration (22.57 ± 2.89) vs. control: (0.96 ± 0.56; *p* = 0.0002, Figs. 1 and 2). Moreover a distinct correlation was observed between the effects of clozapine and haloperidol (*p* = 0.0018). Surprisingly, olanzapine did not affect the aromatase mRNA level (1.18 ± 0.43 vs. control: 0.961 ± 0.56; *p* = 0.99). The aromatase protein levels measured using Western blotting method were in turn not significantly changed after treatment with all studied antipsychotic drugs (olanzapine: 0.83 ± 0.24; clozapine: 0.70 ± 0.18 haloperidol: 0.67 ± 0.15 vs. control 0.62 ± 0.15, *p* > 0.05, Fig. 3).

## Discussion

Although aromatase (CYP19A1) does not take part in the central metabolism of antipsychotic drugs in a direct manner, being a crucial player in the steroidogenesis it may distinctly modify their pharmacological effects in the brain, e.g., by alterations of local Ca^2+^ levels and ionic channels permeability in some neuronal populations. At present, accumulating investigations focus on brain neurosteroid physiology; however, there are no reports dealing with changes in rat brain aromatase expression related to neuroleptic action. Studies on the effects of antipsychotic drugs on central neurosteroid signaling could potentially enlighten mechanistic pathways explaining some of their alternative pharmacological activity.

**Fig. 4:**
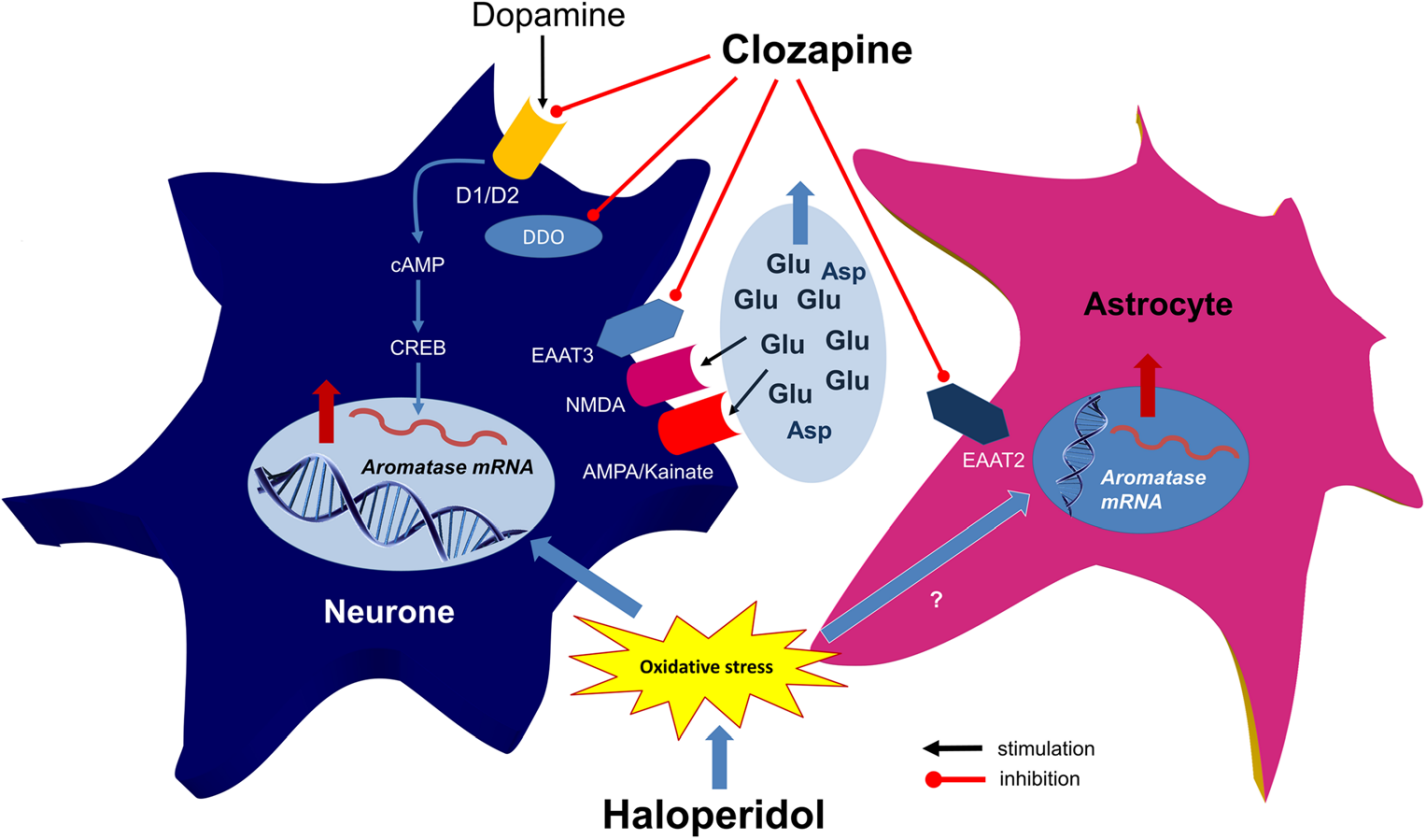
A hypothetical mechanism of the effect of olanzapine and haloperidol on aromatase expression in the rat brain. The CYP19A1 expression in some neurons can probably be modulated by their glutamate NMDA and AMPA/kainate receptors. Clozapine may increase glutamate/aspartate concentration through the inhibition of excitatory amine acids transporters 2 and 3 (EAAT2/EAAT3) and D-aspartate oxidase (DDO) in neurons and astrocytes. Haloperidol-related oxidative stress may in turn stimulate both neuronal and glial aromatase expression that can possibly be a kind of protective mechanisms against drug toxicity. NMDA, N-methyl-D-aspartate glutamate receptor; AMPA, α-amino-3-hydroxy-5-methyl-4-isoxazolepropionic acid glutamate receptor; D1,D2 dopamine receptors; cAMP, cyclic AMP, CREB, cAMP response element binding protein

An observed increase of aromatase mRNA expression after long-term clozapine administration may correspond only partially and indirectly with the previous studies reporting an acute elevation of brain 3α,5α-tetrahydroprogesterone (3α,5α-THP), allopregnanolone, and allotetrahydrodeoxycorticosterone (THDOC) levels in rats acutely treated with clozapine and olanzapine (Barbaccia et al. 2001; Marx et al. 2006). On the other hand, extended administration of clozapine in drug-resistant schizophrenic patients did not affect circulating levels of the 3α,5α-THP and THDOC (Monteleone et al. 2004). In our study, surprisingly, no changes in aromatase mRNA level after treatment with olanzapine occurred, contradicting previous studies (Barbaccia et al. 2001; Marx et al. 2006). The mechanism of clozapine action at the level of aromatase-expressing neurons is not clear. Several possibilities can be considered to explain how this antipsychotic modulates neuronal aromatase production (Fig. 4). Hypothetically, an increase in aromatase mRNA expression may be caused by blockade of selective dopamine receptors located in certain neuronal populations. However, pharmacological study, by Xing et al. 2016, on cultured stem–like radial glial cells (RGCs) showed a cAMP-dependent upregulation of aromatase B mRNA expression after D1 receptor activation by the selective agonist (flupentixol). Moreover, in vitro studies showed that brain aromatase activity was inhibited by both D1/D2 agonists (apomorphine, RU-24213) and antagonists (sulpiride, spiperone, pimozide) (Absil et al. 2001), suggesting that the observed effects are not mediated through binding to dopamine receptors. Because the presence of glutamate AMPA/kainate and NMDA receptors in the aromatase-expressing cells has been confirmed (Balthazart et al. 2003), a glutamatergic hypothesis of clozapine action may also be suggested. Clozapine may enhance neurotransmitter action by increasing its synaptic concentration via aforementioned EAATs’ or DDO blockage (Krzystanek et al. 2015; Sacchi et al. 2017), rather than through binding to glutamate receptors (Barygin et al. 2017). Nevertheless, pharmacological stimulation with glutamate agonists caused an elevation in neuronal calcium levels that quickly depresses the aromatase activity in rodent hypothalamic explants (Balthazart et al. 2006), which is conflicting to the mechanism proposed above. There are also reports that hippocampal aromatase activity and local synthesis of estradiol or pregnenolone may be stimulated by NMDA-dependent calcium influx into the neuroplasm (Hojo et al. 2004; Shibuya et al. 2003). Neurosteroids may therefore act as paracrine modulators of the neural transmission, thereby regulating memory processing in the hippocampus.

Neuronal aromatase activity may play an important role in the putative neuroprotective action of estrogens. An increase of aromatase activity was reported in the rat hippocampus, striatum, and cortex after both toxic and mechanical brain injury (Saldanha et al. 2009). In the present study, long-term treatment with haloperidol increased CYP19A1 mRNA expression in whole brain homogenates. Haloperidol has numerous neurotoxic properties (Nasrallah and Chen 2017; Isom et al. 2013), e.g., inducing disturbances in the oxidative-antioxidative balance (Raudenska et al. 2013). Long-term treatment with haloperidol distinctly modulated the activity of antioxidant enzymes in the rat brain, which correlated with the level of lipid peroxidation (Pillai et al. 2007). An extended haloperidol administration disturbed glutamatergic transmission in the rat prefrontal cortex, that was mainly an effect of NMDAR activity inhibition caused by decreased NR1 and NR2A but not NR2B subunit expression (Fumagalli et al. 2008; Leveque et al. 2000). Possibly, the elevation of aromatase mRNA expression observed in our study may be part of a neuronal protective mechanism against an extended treatment with this drug (Fig. 4). An increase of aromatase mRNA level was not reflected in alterations of protein expression in whole brain homogenates. We cannot therefore precisely estimate to what extent the changes observed affected the potential local synthesis of neuroactive estrogens. The particular brain regions and neuronal assemblies may probably represent different patterns of CYP19A1 protein synthesis after neuroleptic pharmacomodulation. To sum up, antipsychotics may affect aromatase expression in the rat brain, which may be one of the alternative ways of their action in the CNS. A regulatory impact on brain steroidogenesis may be one of the clinically important, so far unknown, possibly sex-dependent mechanisms of pharmacological effects triggered by both typical and atypical neuroleptics.

It should be pointed out that there are some limitations to our study. For instance, the mRNA and protein levels in the precisely defined brain structures instead of the whole hemispheres were not measured, and the estrogen concentrations were not determined and the precise analysis of signaling pathways was not provided.

Nevertheless, this new finding represents an initial introduction to forthcoming experimental works on the relationships between neuroleptic action and brain steroidogenesis in animal model. Undoubtedly, these initial data require further basic pharmacological, biochemical, and behavioral studies on the wider spectrum of antipsychotic drugs.
